# Corticosteroid prescribing in palliative care settings: a retrospective analysis in New Zealand

**DOI:** 10.1186/1472-684X-13-7

**Published:** 2014-03-08

**Authors:** Anne Denton, John Shaw

**Affiliations:** 1 School of Pharmacy, Faculty of Medical and Health Sciences, The University of Auckland, Building 505, 85 Park Road, Auckland, Grafton, New Zealand

**Keywords:** Palliative care, Hospices, Corticosteroids, Prescribing, Evidence-based medicine, Guidelines

## Abstract

**Background:**

Corticosteroids are a potent group of medicines, with many adverse effects, that are widely prescribed in palliative care for both specific and non-specific indications. The aim of this study was to document current patterns of corticosteroid prescribing in New Zealand palliative care settings and to reflect on whether they were in line with international experience.

**Methods:**

A retrospective review of inpatient use of corticosteroids was undertaken in a sample of six New Zealand hospices. Data were collected on numbers of patients prescribed corticosteroids, indications for use, choice of agent, doses and dosage changes, duration of course, incidence of adverse effects, method of stopping, use of guidelines, and processes for monitoring and review.

**Results:**

The case notes of 1179 inpatients were reviewed and 768 patients (65.1%) had received at least one course of corticosteroids. There was a marked consistency in the proportion of patients prescribed corticosteroids among the sample hospices (61-69%). Detailed information was recorded for a sample of 260 patients. Corticosteroids were prescribed most commonly for non-specific reasons (40.4% of prescribing events), followed by neurological (25.3%) and soft tissue infiltration symptoms (14.4%). The agent of choice was dexamethasone with a dose range of 1 mg to 40 mg and a median dose of 8 mg. The median course duration for all corticosteroid prescribing events was 29 days. Abrupt stopping occurred in 72 (23.2%) cases, of these 35 (49%) had been on a course of corticosteroids for more than three weeks. Guidelines were only available in one hospice. Monitoring and review was documented in 135 (52%) of cases, and adverse effects were recorded in 82 (32%); these are likely to be underestimates due to a high level of non-recording.

**Conclusions:**

This New Zealand study showed that corticosteroids are widely prescribed in palliative care, most commonly for non-specific indications. These findings are consistent with the international literature in this area and this large, multi-site study adds weight to the findings and the need for ongoing discussion about the place of these drugs in palliative care.

## Background

Corticosteroids can be considered to be ‘old’ medicines; their prescribing in palliative therapy has been commonplace since the late 1950s [1-3]. They are commonly prescribed for patients in palliative care for both specific and non-specific indications [4-9]. Corticosteroids are potent medicines with frequent adverse effects and while the intent is to achieve beneficial results for the patient, the consequences of long-term use may counteract this objective [10]. There have been few randomised controlled trials of corticosteroids in palliative care [7,11-13], and caution has been voiced about their haphazard use [14]. Corticosteroid use short-term for specific reasons, such as neurological (spinal cord compression) or soft tissue infiltration (e.g. by abdominal tumours), is supported by weak evidence [7,15].

Concerns have been expressed about the non-specific use of corticosteroids, the most common indication for their prescribing, since there appears to be little robust evidence to support this practice [5]. Some authors have observed that the use of corticosteroids for ‘non-specific’ symptoms (e.g. appetite loss, nausea, fatigue, pain, shortness of breath or poor wellbeing) had some positive short-term results, but this benefit seldom lasted more than four weeks [14]. Several studies reported a significant improvement in appetite and strength after two weeks of dexamethasone, but declining effects after four weeks [16,17]. Some caution has to be exercised in interpretation of the literature as there are differences in terminology with respect to both palliative care patients (some corticosteroid studies relate only to ‘advanced cancer’ or ‘pre-terminal’ cancer) and to the categorisation of corticosteroid indications, particularly the definition of ‘non-specific’ symptoms. Some authors restrict the definition of non-specific to anorexia-cachexia symptoms (anorexia, fatigue, weight loss), while most have included more general symptoms such as breathlessness, nausea and pain. Pain, of course, could also be attributable to specific causes. The broader definition of non-specific is used in this paper.

Twycross [18] advocated the use of a clear plan when corticosteroids were being administered, including regular monitoring leading to dose adjustment, and prescribing for the shortest time frame at the lowest effective dose [18]. Other authors recommended that if corticosteroids were of no benefit then they should be discontinued [6,10]. Lundstrom et al. [19] argued that the benefits of high-dose corticosteroids in the dying patient, while controversial, may have a profound effect on end of life symptoms and perceptions of both patients and families in terms of hope and psychological well-being, even if short-term. They commented that “reduced symptoms contribute to feelings of normalising life, symbolising hope” [19].

The main synthetic corticosteroids used in palliative care are prednisone, dexamethasone, methylprednisolone and betamethasone [5,20-22]. Based on their chemical structures [23], some corticosteroids have stronger glucocorticoid properties which confer greater anti-inflammatory effects, but also a greater likelihood of adverse effects [24]. The long-term administration of glucocorticoids can lead to Cushing’s syndrome [25,26], the consequences of which can be of such significance with respect to changes in physical appearance and emotional state that some patients and families consider the effects of long-term corticosteroids to be worse than the original indication [14,27-29].

All four agents have long biological half-lives, particularly dexamethasone and betamethasone [24]; they can therefore be given as a once daily dose, early in the day [30-32]. Internationally, the corticosteroid of choice appears to be dexamethasone [11,14,20,33-38], which, with betamethasone, has the highest anti-inflammatory properties [24]. Reasons for its choice may include the availability of oral and parenteral formulations, lower cost, longer duration of action, lack of mineralocorticoid effects and higher glucocorticoid activity leading to an enhanced anti-inflammatory effect [20,24]. Many studies have found that a high proportion of patients in a palliative care service (32-80%) will receive at least one course of corticosteroids [4,6,8,14,28,34,35,39-42]. The literature is also consistent that 50% to 70% of patients prescribed corticosteroids for more than three weeks will have adverse effects [3,4,14]. These effects are not always recognised as being corticosteroid in origin and may be wrongly assumed to be part of the dying process [16].

Shafford [14] advocated the development of guidelines to curb the potential for haphazard prescribing [14]. Guidelines need to be evidence-based to ensure the best outcome for the patient with minimum adverse effects, as well as being easy to follow, clinically relevant, comprehensive, and flexible for busy clinicians [5]. A number of studies and guidelines advise that if corticosteroids show no benefit they should be stopped before adverse effects occur [6,10,18,42-44], but a course taken for longer than three weeks (some guidelines suggest two weeks) should be reduced gradually and not stopped abruptly [14]. Abrupt cessation may lead to adrenal suppression and an increase in terminal restlessness [25,43,45-47].

Given the challenges associated with the prescribing of these agents in palliative care patients, and evidence in the literature of sub-optimal practice, it was considered timely to undertake a review of their prescribing in a New Zealand context. The aim of this study was to document current patterns of corticosteroid prescribing in a sample of New Zealand palliative care settings and to reflect on whether it is in line with international experience.

## Methods

This study was approved by the New Zealand Multi-regional Ethics committee (MEC/08/37/EXP). It was accomplished by undertaking a retrospective review of corticosteroid prescribing in a sample of New Zealand hospices over a defined period (January 1st to December 31st 2007). The study was the first phase of a larger study investigating influences on the prescribing of corticosteroids in the palliative care setting. The second phase which investigated clinicians’ perspectives on corticosteroid prescribing has been reported elsewhere.

### Study sites

In 2007, there were 32 hospices throughout New Zealand. A purposive sample of six hospices (approximately one in five) was considered sufficient to give an accurate ‘snapshot’ of corticosteroid prescribing at the time. Characteristics of the sample hospices are shown in Table 1. The rationale was to have a balance of larger urban and smaller rural hospices, thus gaining a cross-section of corticosteroid prescribing nationally. The invitation to be part of this review was accepted by the hospice managers and medical directors. Each hospice was assigned a code number from one to six for confidentiality purposes. Individual patient consent forms were not required because patient data was anonymous (all patients had died by the time the review was undertaken).

**Table 1:** Characteristics of sample hospices

**Location**	**Population base**	**Number of beds**
**Hospice 1:** Urban	295,000	18
**Hospice 2:** Urban with large rural catchment	180,000	12
**Hospice 3:** Urban	102,000	10
**Hospice 4:** Urban with rural catchment	60,000	5
**Hospice 5:** Urban with large rural catchment	104,000	6
**Hospice 6:** Metropolitan	426,000 (not sole provider)	9

### Sample size

A pilot study was performed at the researcher’s (AD) home hospice to estimate the proportion (%) of inpatients likely to be prescribed corticosteroids. This hospice was not included in the study sample because of the close working relationship with the author. Of 250 inpatients at this hospice, 49% had been prescribed corticosteroids. Using this information, a sample of approximately 1250 inpatients was considered sufficient to enable estimation of the true proportion of palliative care patients prescribed corticosteroids throughout New Zealand, with a 95% confidence level of ± 2.8%. For the patients prescribed corticosteroids whose records were examined, a sample size of 210 was considered sufficient to enable an estimation of a true proportion of patients with a drug review, with a 95% confidence interval of 6.1%. The author (AD) initially reviewed 1179 individual inpatients’ case notes from the six hospices for the 2007 calendar year and separated out the notes for all those who had been prescribed at least one course of corticosteroids. For the 768 patients who had been prescribed corticosteroids, every third set of notes was selected for review and corticosteroid prescribing details were entered into a database to allow further analysis.

### Data collection

Data were collected between February and December 2009 from the 2007 inpatient case notes of those patients prescribed corticosteroids selected as part of the study sample. Data on survival times beyond 2007 were not recorded, but by the time the case notes were reviewed in 2009, all patients in the sample had died (it was an Ethics requirement that only deceased patients’ notes could be accessed). The database recorded patient demographics and confirmed diagnoses, as well as details of each corticosteroid prescribing event and corticosteroid review (Table 2).

**Table 2:** Corticosteroid prescribing and review database

**Corticosteroid prescribing and review database**	
1	Hospice code
2	Individual patient code
3	Patient history: gastrointestinal tract cancer; urogenital cancer; lung cancer; breast cancer; melanoma; haematological cancers; brain cancer; other cancers; non-cancer
4	Patient age (years)
5	Patient gender
6	Corticosteroid prescribing event: which agent was prescribed? (separate entry for each prescribing event)
7	Date, if noted, when the corticosteroid was commenced
8	Initial dose of corticosteroid prescribed
9	Route: oral, subcutaneous; intramuscular; intravenous
10	Prescribed by: hospital doctor; general practitioner; hospice doctor
11	Indication (reason) for corticosteroid prescribing (see Table 3)
12	Concurrent prescribing of: omeprazole; NSAID; phenytoin; zopiclone
13	Corticosteroid stopped: gradually; abruptly; patient died while still on their corticosteroid
14	Adverse effects to corticosteroids: yes; no; not recorded
15	Was drug reviewed?
16	Date of review
17	Was there an indication change for giving corticosteroid: if yes, complete a new entry
18	Dose change decision: decrease dose; increase dose; stop corticosteroid; no change; change corticosteroid: if yes, complete a new entry
19	Reason for change: patient deterioration; no change in patient; improvement in patient; not recorded

If a patient was prescribed a different corticosteroid as the result of a drug review, or if a corticosteroid was prescribed for a new indication, this was recorded as a separate prescribing event. The sample included both patients who continued a course of corticosteroids started before admission and those who commenced corticosteroids after admission. Multiple re-admissions were common; these were identified through the case notes and patients retained the original research code allocated to them. If the patient was still on a course of corticosteroids from a previous admission, this was recorded as a continuation event; if a new course was prescribed, this was recorded as a separate prescribing event.

Eight indications were selected for this study (Table 3). While some of these are not mutually exclusive (e.g. soft tissue infiltration can include capsular stretching by liver metastases and so on) and the definition of non-specific is open to interpretation, the indications listed were similar to those used in a number of previous studies so as to allow useful comparisons. The category of not clear/other was only used when there was an indication which could not be otherwise classified, or where there was insufficient information concerning the indication in the patient notes. Stopping abruptly was defined as the patient being on a corticosteroid one day and not the next; drug review was defined as being performed if it was recorded in the patient notes; and adverse effects were defined as those listed as corticosteroid-specific in standard pharmacology texts, notably the appearance of Cushingoid features [23-26].

**Table 3:** Description of indications

	
1	Non-specific: to include lack of appetite, wellbeing, fatigue nausea, vomiting, pain and shortness of breath
2	Neurological to include: raised intracranial pressure, cerebral tumours, spinal cord compression and nerve compression or infiltration
3	Capsular stretching: to include liver metastases and other visceral organ metastases
4	Soft tissue infiltration: to include head and neck tumours and abdominal and pelvic tumours
5	Tenesmus: rectal pain due to invasive tumours
6	Inflammation with syringe driver sites (subcutaneous route)
7	Not clear/other: to include any indication, which was either not clear or did not fit in the other categories
8	Chemotherapy

### Data analysis

Data are presented as descriptive statistics and frequencies with 95% confidence intervals, means and standard deviations (s.d.), medians and interquartile ranges (IQR) were used as appropriate to describe the characteristics of the patients and their treatments. Chi-square tests were performed to test for differences in the proportions of patients receiving particular treatments between hospices. Only those situations where a statistically significant result was found are reported. The clustering of data where patients had a number of dose changes within one corticosteroid prescribing event was allowed for in the analyses and calculation of confidence intervals. SAS version 9.12 was used in the analyses of the data. As the hospices were not randomly selected, they were included as fixed factors in the analyses.

## Results

At the time of this retrospective review, 1179 inpatient notes were reviewed. As shown in Table 4, at least one course of corticosteroids was prescribed for 768 of these patients. There was a marked consistency in the proportion of patients prescribed corticosteroids across the six hospices, ranging from 61% to 69% with a mean of 65.1% (s.d. 3.3%); these differences were not significant (p = 0.366). Of the 768 patients prescribed corticosteroids, one in three (260) were selected for detailed analysis. For the 260 patients reviewed, there were 312 corticosteroid prescribing events recorded during the study period. As would be anticipated, there were numerous dose changes and some route changes during a single course of corticosteroids. There were 891 such changes documented in the patient notes; however, this is likely to be an underestimate as it was very difficult to track this information in a number of cases.

**Table 4:** Patients prescribed corticosteroids in the sample hospices

**Facility**	**Number (%) of patients at each hospice prescribed at least one course of corticosteroids**
**Number of patients**	
**Hospice 1**	
297	204 (68.7%)
**Hospice 2**	
235	144 (61.3%)
**Hospice 3**	
175	120 (68.6%)
**Hospice 4**	
111	71 (64.0%)
**Hospice 5**	
142	94 (66.2%)
**Hospice 6**	
219	135 (61.6%)
**Total**	
**1179**	**768 Mean (s.d.): 65.1% (3.3%)**

The demographic characteristics of those patients prescribed corticosteroids whose case notes were reviewed (260) are shown in Table 5. A small number of patients (6) had a second cancer diagnosed during the study; to avoid confusion, Table 5 lists only the first diagnosis recorded. As shown in Table 6, the three most common indications for corticosteroids were non-specific symptoms, neurological symptoms and soft tissue infiltration which accounted for 80% of all prescribing events. The most common indication was non-specific, ranging from 33% to 61% of total prescribing events across the six hospices. The difference between hospices for non-specific prescribing was statistically significant (*p* = 0.026), but this has to be interpreted with caution due to possible different interpretations of non-specific. There were 30 prescribing events (9.6%) classified as not clear/other.

**Table 5:** Demographic characteristics of sampled patients prescribed corticosteroids

**Facility (Number of patients)**	**Cancer diagnosis number of patients (%)**		**Gender number of patients (%)**		**Age (Years)**
	**Yes**	**No**	**Male**	**Female**	**Mean (s.d.)**
**Hospice 1** (70)	66 (94%)	4 (6%)	28 (40%)	42 (60%)	66 (14)
**Hospice 2** (50)	50 (100%)	0 (0%)	28 (56%)	22 (44%)	66 (13)
**Hospice 3** (40)	36 (90%)	4 (10%)	18 (45%)	22 (55%)	71 (12)
**Hospice 4** (24)	21 (87%)	3 (13%)	12 (50%)	12 (50%)	68 (13)
**Hospice 5** (31)	31 (100%)	0 (0%)	11 (35%)	20 (65%)	63 (14)
**Hospice 6** (45)	44 (98%)	1 (2%)	22 (49%)	23 (51%)	62 (13)
**Total (260)**	248* (95%)	**12 (5%)**	**119 (46%)**	**141 (54%)**	**66 (13)**

*A small number of patients (6) had a second cancer diagnosed during the course of the study; only the initial diagnosis is listed in this table.

**Table 6:** Corticosteroid prescribing events by indication in the six sample hospices

**Indication**	**Number of prescribing events (%)**	**95% Confidence limits**
1. Non-specific	126 (40.4%)	34.6% – 46.1%
2. Neurological:	79 (25.3%)	20.3% – 30.3%
3. Capsular stretching	14 (4.5%)	2.2% – 6.8%
4. Soft tissue infiltration	45 (14.4%)	10.4% – 18.4%
5. Tenesmus	3 (1.0%)	0.0% – 2.0%
6. Inflammation with syringe driver sites	10 (3.2%)	1.3% – 5.1%
7. Not clear/other	30 (9.6%)	6.2% – 13.0%
8. Chemotherapy	5 (1.6%)	0.0% – 3.2%
**Total**	312* (100%)	

*There were 312 separate corticosteroid prescribing events documented in the case notes of the 260 patients reviewed.

Dexamethasone was by far the most commonly prescribed agent (72.7% of all prescribing events) and both the oral and parenteral routes were used. It was the only medicine to be prescribed for all eight indications. Prednisone and methylprednisolone were prescribed in 21.5% and 5.8% of instances respectively. The two indications with the highest proportion of dexamethasone prescribing were non-specific and neurological symptoms (29.9% and 31.6% of dexamethasone events respectively).

Table 7 illustrates the dose ranges for the three agents by indication, including the median starting and final doses. An attempt was made to determine the cumulative dose per patient, but this was not possible to accurately record for most patients. There was little variation in the median doses of the agents across the six hospices, particularly for the main indications, while the median dose varied according to indication in some instances. In the case of dexamethasone, for example, the median dose was 4 mg for non-specific indications and 8 mg for neurological indications.

**Table 7:** Dose ranges of corticosteroids prescribed by indication

**Drug prescribed by indication (Number of prescribing events)**	**Median (IQR) starting dose mg**	**Dose range mg**	**Median (IQR) final dose mg**
**1. Non-specific**			
Pred (59)	20 (20)	5 - 60	20 (20)
Meth (3)	125 (0)	125 - 125	125 (0)
Dex (67)	4 (4)	1 - 8	4 (4)
**2. Neurological**			
Pred (0)	-	-	-
Meth (6)	125 (0)	80 - 125	125 (0)
Dex (71)	8 (8)	2 - 16	8 (8)
**3. Capsular stretching**			
Pred (1)	20 (0)	20 - 20	20 (0)
Meth (0)	-	-	-
Dex (12)	8 (4)	4 - 12	8 (4)
**4. Soft tissue infiltration**			
Pred (1)	20 (0)	20 - 20	20 (0)
Meth (7)	125 (0)	125 - 125	125 (0)
Dex (32)	8 (4)	4 - 16	8 (4)
**5. Tenesmus**			
Pred (0)	-	-	-
Meth (0)	-	-	-
Dex (2)	8 (0)	8 - 8	8 (0)
**6. Inflammation s/c sites**			
Pred (0)	-	-	-
Meth (0)	-	-	-
Dex (10)	0.75 (0.5)	0.5 - 1	1 (0.5)
**7. Not clear/other**			
Pred (3)	10 (0)	6 - 80	10 (0)
Meth (1)	1000** (0)	1000 - 1000**	1000** (0)
Dex (24)	8 (4)	1 - 32	4 (4)
**8. Chemotherapy**			
Pred (2)	15(0)	10 - 20	15 (0)
Meth (1)	125 (0)	125 - 125	125 (0)
Dex (6)	12 (8)	4 - 16	8 (6)
Total*			
**Pred (66, 21.5%)**	**20 (20)**	**5 - 80**	**20 (20)**
**Meth (18, 5.8%)**	**125 (0)**	80 - 1000**	**125 (0)**
**Dex (224, 72.7%)**	**8 (4)**	**1 - 40**	**8 (4)**

Key: IQR: Interquartile range; Pred: prednisone; Meth: methylprednisolone; Dex: dexamethasone.

*308 of 312 corticosteroid prescribing events are reported (four missing sets of data).

**There was a single instance of methylprednisolone being prescribed at a dose of 1000 mg; the reason for this dose was not recorded in the notes.

The length of time patients were prescribed corticosteroids varied from a single dose prescribed for one day, to a course continuing for 477 days, with a median duration for all indications of 29 days (interquartile range (IQR): 20 days) as shown in Table 8. It should be noted that full information about course duration was available for only 214 of the 312 corticosteroid prescribing episodes (68.5%), due to difficulty in interpretation of records or lack of recording in the notes. A separate analysis of this data showed that 119 of 212 patients (56.1%) received a corticosteroid course for longer than three weeks (at which time some degree of adrenal suppression might be anticipated). Data were collected on the manner in which corticosteroids were stopped, that is whether they were reduced gradually, stopped abruptly, or the patient died while on a course of corticosteroids. Seventy-two of 310 courses were stopped abruptly (23.2%) and a corticosteroid had been prescribed for longer than three weeks in 35 of these cases (49%). Table 9 lists the reasons for abrupt stopping; it is notable that in nearly half of the documented instances (45.8%), the patient was unable to swallow.

**Table 8:** Duration of corticosteroid course by indication

**Indication (Number of prescribing events)**	**Duration of course (days)**		
	**Minimum**	**Maximum**	**Median (IQR)**
1. Non-specific (83)	1	432	28 (22)
2. Neurological (64)	2	477	34 (18)
3. Capsular stretching (9)	2	312	18 (23)
4. Soft tissue infiltration (39)	2	101	14 (10)
5. Tenesmus (2)	4	7	5.5 (0)
6. Inflammation s/c sites (1)	1	1	1 (0)
7. Not clear/other (16)	3	172	7 (6)
8. Chemotherapy (0)	-	-	-
Total (214)*	**1**	**477**	**29 (20)**

Key: IQR: Interquartile range.

*The full duration of the corticosteroid course was able to be determined for 214 of the 312 corticosteroid prescribing events.

**Table 9:** Reasons for stopping corticosteroid abruptly

**Reason for stopping corticosteroid abruptly**	**Number of events (%)**
1. Patient not swallowing	33 (45.8%)
2. Patient stopped their own corticosteroid	4 (5.6%)
3. Gastric bleed	2 (2.8%)
4. Documented adverse effects	3 (4.2%)
5. Switched to bolus dose or short course	6 (8.3%)
6. Reason not documented	24 (33.3%)
**Total**	72* (100%)

*72 of 310 corticosteroid prescribing events (23.2%) were recorded as ‘stopped abruptly’.

The proportion of patients with adverse effects to corticosteroids recorded in their notes ranged from 15% to 45% across the six hospices.. It is highly likely that more patients experienced adverse effects than were recorded. A significant difference in recording between the hospices was found (*p* = 0.0001). While the lack of recording is a notable finding in itself, it is not possible to draw useful conclusions about the true incidence of adverse effects from this data.

Table 10 illustrates that documented reviews of corticosteroid prescribing varied considerably between the hospices with percentages ranging from 28% to 66% with a mean (s.d.) of 52% (13.1%). Guidelines were evident in only one hospice (Hospice 3). Each patient in this hospice had a separate corticosteroid sheet on which the reducing corticosteroid regimen was written. The medication chart, when referring to the corticosteroid, stated ‘as per protocol’. As there were often several tapering schedules on separate forms for the same patient running concurrently, sometimes with contradictory information, this data was difficult to interpret retrospectively. The recorded reviews in the notes are probably an underestimate of actual reviews at this hospice.

**Table 10:** Documented corticosteroid reviews

**Facility (Number of patients)**	**Number of reviews documented (%)**
**Hospice 1** (70)	46 (65.7%)
**Hospice 2** (50)	28 (56%)
**Hospice 3** (40)*	11* (27.5%)
**Hospice 4** (24)	11 (45.8%)
**Hospice 5** (31)	14 (45.2%)
**Hospice 6** (45)	25 (55.5%)
**Total (260)**	**135 mean (s.d.): 52% (13.1%)**

*Hospice 3 used a separate corticosteroid dosing sheet on which the reducing regimen was written; as a consequence, a smaller percentage of reviews were documented in the notes.

## Discussion

While there have been a number of reports of corticosteroid prescribing practices in the palliative care setting internationally, this retrospective review of corticosteroid prescribing across six hospices is, to our knowledge, the first of its kind in New Zealand. It also appears to be one of only a few multi-site retrospective analyses to be reported and is possibly the largest of its kind to date. It mostly confirms previous literature findings, as well as raising some specific issues, such as abrupt stopping, for further debate.

The proportion of inpatients prescribed corticosteroids in this study (65%) was consistent with previous literature (4,6,8,14.28,34,35,39-42), and there was little variation between the hospices (range 61 – 69%) which suggests that this is an accurate reflection of prescribing practice throughout New Zealand. The most frequent prescribing indication was non-specific (40.4%), again this is in line with international experience if a broad definition of non-specific is used [4,14]. While this is a notable result, given that there is relatively little published evidence to support the use of corticosteroids for non-specific reasons [48], it has to be interpreted with some caution because of varying definitions of non-specific indications in the literature. In addition, a recent randomised controlled trial of dexamethasone 8 mg daily has demonstrated significant reductions in cancer-related fatigue in patients with advanced cancer, providing good evidence for its use in this non-specific indication [49]. Neurological (25.3%) and soft tissue infiltration (14.4%) were the most frequent specific indications; this finding is supported by two UK studies [40,43].

The only corticosteroid prescribed for all indications was dexamethasone (72.7% of prescribing events), with less prescribing of prednisone (21.5%) and methylprednisolone (5.8%). Dexamethasone is currently the most commonly used corticosteroid internationally [11,14,20,33-38], principally because it has the greatest anti-inflammatory effect and the longest biological half-life [24]. Whilst corticosteroid guidelines were only evident in one of the sample hospices studied, it was reassuring to discover that, without guidelines, the median dose for the most common indications were similar. This was contrary to Shafford’s [14] study that suggested there was a divergence of dose ranges even in the presence of guidelines [13]. However, prescribers in the current study seldom used the same course duration or rate of reduction of dose. It is interesting to speculate as to whether guidelines are required in the New Zealand context, given the notable consistency in the proportion of patients receiving corticosteroids and in the doses used for different indications. Conversely, the wide variation in dose reduction or stopping, supports guideline development and use.

A number of researchers suggest that corticosteroids can be stopped abruptly if they have been prescribed for less than three weeks [50], but should be titrated down after this period. In this study, 72 (23.2%) patients had their corticosteroids stopped suddenly and 35 of these (49%) had been on the agent longer than three weeks, including those whose medicines were stopped when they could no longer swallow. Some literature suggests that stopping long-term corticosteroid therapy abruptly is unethical and may lead to an adrenal crisis, restlessness, anxiety and hasten death [25,43,45-47]. Although stopping long-term therapy if the patient is no longer swallowing appears to be common practice, it is both clinically and ethically questionable and merits further debate, particularly as parenteral formulations of these medicines are available and a route change is possible.

In the sample hospices, monitoring and review could not be assumed to have taken place if it had not been recorded in the patient notes. It was not clear if this was a case of reviews and monitoring being conducted and not recorded, or not occurring at all. Lack of recording of adverse effects of corticosteroids in patient notes was evident. These effects appeared to be under-reported (15% to 45% across the hospices). These findings are supported by a 2008 nationwide survey in Japan where a low percentage of adverse effects was also reported [6], yet a number of authors suggest that 50% to 75% of patients prescribed corticosteroids for more than three weeks will have adverse effects [3,4,14].

There were a number of limitations to the current study. The study sample was restricted to New Zealand, so generalizability of findings internationally may be limited. Nevertheless, the findings generally mirrored overseas experience as reported in the literature. The ‘snapshot’ of corticosteroid prescribing was for the 2007 calendar year and there may have been practice changes, such as the introduction of guidelines, since that time. As the review was retrospective and all patients had died by the time data was collected (2009), it was not possible to follow-up patients to corroborate findings.

Accessing and interpreting case notes were often challenging and each hospice had a different approach to where and how prescribing information was recorded. As the only information available to the researchers was inpatient notes and associated documentation such as drug charts and correspondence, it is reasonable to speculate whether differences in the individual hospices were due to actual prescribing or to recording practices. The authors made every attempt to minimise the impact of different recording practices (the majority of records were handwritten).

Some data was very difficult to retrieve or interpret using the retrospective analysis of case notes. For example, it was not possible to determine cumulative corticosteroid doses for the majority of events, nor was it possible to accurately track dose or route changes, particularly for tapering regimens. Additionally, it is highly likely that reviews and adverse effects are underestimated in this study, because of non-recording. Given the above described limitations of a retrospective design it would be useful to conduct a prospective study in actual hospice settings. Notwithstanding these limitations, the study has provided some useful insights into the prescribing of corticosteroids in the context of palliative care.

## Conclusion

The proportion of New Zealand palliative patients prescribed corticosteroids was in line with international literature, but there was a notable consistency between hospices in this study. Most commonly, corticosteroids were prescribed for non-specific reasons and the agent of choice was dexamethasone. Dose ranges and course durations largely mirrored international experience. Only one hospice used guidelines and most prescribing appeared to be prescriber-driven. The reduction and stopping of corticosteroids appeared to be *ad hoc* even after long-term use, and there was a general lack of documentation and recording of review and monitoring of these drugs and their adverse effects. These findings are generally consistent with the international literature in this area and this study adds weight to the need for ongoing discussion about the role of these drugs in palliative care.

## Competing interests

The authors declare that they have no competing interests.

## Authors’ contributions

AD and JS were involved in the conception, design and implementation of the research. AD undertook the data collection and analysis. AD and JS were both involved in data interpretation, drafting of the paper and in the final review and approval. AD was formerly a specialist palliative care pharmacist in a New Zealand hospice. Both authors read and approved the final manuscript.

## Pre-publication history

The pre-publication history for this paper can be accessed here:

http://www.biomedcentral.com/1472-684X/13/7/prepub
